# Exploring the causal effect of placental physiology in susceptibility to mental and addictive disorders: a Mendelian randomization study

**DOI:** 10.3389/fpsyt.2024.1396837

**Published:** 2024-07-29

**Authors:** Pablo Jácome-Ferrer, Javier Costas

**Affiliations:** ^1^ Psychiatric Genetics group, Instituto de Investigación Sanitaria de Santiago de Compostela (IDIS), Santiago de Compostela, Spain; ^2^ Universidade de Santiago de Compostela (USC), Santiago de Compostela, Spain; ^3^ Complexo Hospitalario Universitario de Santiago de Compostela (CHUS), Servizo Galego de Saúde (SERGAS), Santiago de Compostela, Spain

**Keywords:** birth weight, depression, fetal neurodevelopment, Mendelian randomization, mental disorders, placental physiology, psychiatric genetics, trophoblast invasion

## Abstract

**Background:**

Epidemiological studies have linked low birth weight to psychiatric disorders, including substance use disorders. Genomic analyses suggest a role of placental physiology on psychiatric risk. We investigated whether this association is causally related to impaired trophoblast function.

**Methods:**

We conducted a two-sample summary-data Mendelian randomization study using as instrumental variables those genetic variants strongly associated with birth weight, whose effect is exerted through the fetal genome, and are located near genes with differential expression in trophoblasts. Eight psychiatric and substance use disorders with >10,000 samples were included as outcomes. The inverse variance weighted method was used as the main analysis and several sensitivity analyses were performed for those significant results.

**Results:**

The inverse variance weighted estimate, based on 14 instrumental variables, revealed an association, after correction for multiple tests, between birth weight and broadly defined depression (β = −0.165, 95% CI = −0.282 to −0.047, *P* = 0.0059). Sensitivity analyses revealed the absence of heterogeneity in the effect of instrumental variables, confirmed by leave-one-out analysis, MR_Egger intercept, and MR_PRESSO. The effect was consistent using robust methods. Reverse causality was not detected. The effect was specifically linked to genetic variants near genes involved in trophoblast physiology instead of genes with fetal effect on birth weight or involved in placenta development.

**Conclusion:**

Impaired trophoblast functioning, probably leading to reduced fetal brain oxygen and nutrient supply, is causally related to broadly defined depression. Considering the therapeutic potential of some agents to treat fetal growth restriction, further research on the effect of trophoblast physiology on mental disorders may have future implications in prevention.

## 1 Introduction

Epidemiological data revealed an association between prenatal/perinatal problems, such as gestational diabetes, gestational hypertension, maternal infections during gestation, nutritional deficits during gestation, or preeclampsia and mental disorders (1–3). These agree with the fetal origins of mental health, framed within the developmental origins of health and disease hypothesis (4). The hypothesis proposes that inappropriate fetal environment may affect brain development, leading to an increased susceptibility to mental disorders later in life.

Low birth weight is commonly used as an easy measure of putative fetal adversity. Meta-analyses of observational studies identified that low birth weight is associated with several mental disorders, such as depression (5), psychosis (2) or autism spectrum disorder (ASD) (3). Large epidemiological studies also reported an association between low birth weight, or small for gestational age, and substance use disorder in adolescence/young adulthood (6) and adults (7, 8). Remarkably, Pettersson et al. (8) have also found an association between low birth weight and a latent variable measuring a general factor of psychopathology, based on 12 outcomes: depression, anxiety, obsessive-compulsive disorder (OCD), post-traumatic stress disorder (PTSD), bipolar disorder (BIP), alcohol abuse, drug use, violent crimes, attention-deficit/hyperactivity disorder (ADHD), ASD, schizophrenia (SCZ), and schizoaffective disorder.

However, association does not imply causality. Mendelian randomization (MR) is a methodological approach to test for causality using genetic predisposition as a proxy for the exposure factor (9). Arafat and Minica (10) performed a two-sample MR study using GWAS for birth weight from the Early Growth Genetics Consortium (EGGC) (11), as a proxy of exposure to fetal adversity, to test the impact of this exposure on mental disorders, specifically, depression, SCZ, and ADHD. They did not find any evidence of causality. Conversely, Orri et al. (12) revealed a putative causal effect of low birth weight on ADHD by MR. This causal effect was not detected for other mental disorders, such as BIP, SCZ, or depression. One explanation for the contradictory results of ADHD may be that while Arafat and Minica (10) used unadjusted SNP effects, Orri et al. (12) used direct fetal effects adjusted for maternal genotype.

Although easy to measure, birth weight has limitations as a proxy for fetal adversity. It does not distinguish between normal growth in constitutionally small but healthy newborns and fetal growth restriction, the condition in which a fetus does not reach its growth potential (13, 14). A key player in fetal growth restriction is the placenta. The main cell type of placenta is the trophoblast, whose origin is fetal. Villous cytotrophoblasts are stem cells that give rise to syncytiotrophoblast and extravillous trophoblasts. Syncytiotrophoblasts are involved in most functions of the placenta, such as active transport of nutrients, waste excretion, oxygenation, hormone production, and protection against xenobiotics and the maternal immune system. Extravillous trophoblasts are involved in the invasion of maternal decidua to establish the maternal-fetal circulation through vascular remodeling (15–17).

A role of placenta on risk to psychiatric disorders has been suggested by an interaction between polygenic risk scores (PRS) of SCZ and obstetric complications in the onset of SCZ (18). Furthermore, this interaction was mainly due to PRS of genes highly expressed in placenta and differentially expressed in placenta of complicated pregnancies, referred to as PlacPRS. The interaction, restricted to birth asphyxia, has been confirmed in a Norwegian sample (19). However, it was not replicated in a larger independent study (20). The lack of replication may be due to a type I error or to the use of different scales to measure obstetric complications at each study (12, 21). PlacPRS was also negatively associated with neonatal brain volume and cognitive development at one year (22). Interestingly, the Norwegian study also detected an interaction between PlacPRS and birth asphyxia in neonatal head circumference (19).

**Figure 1:**
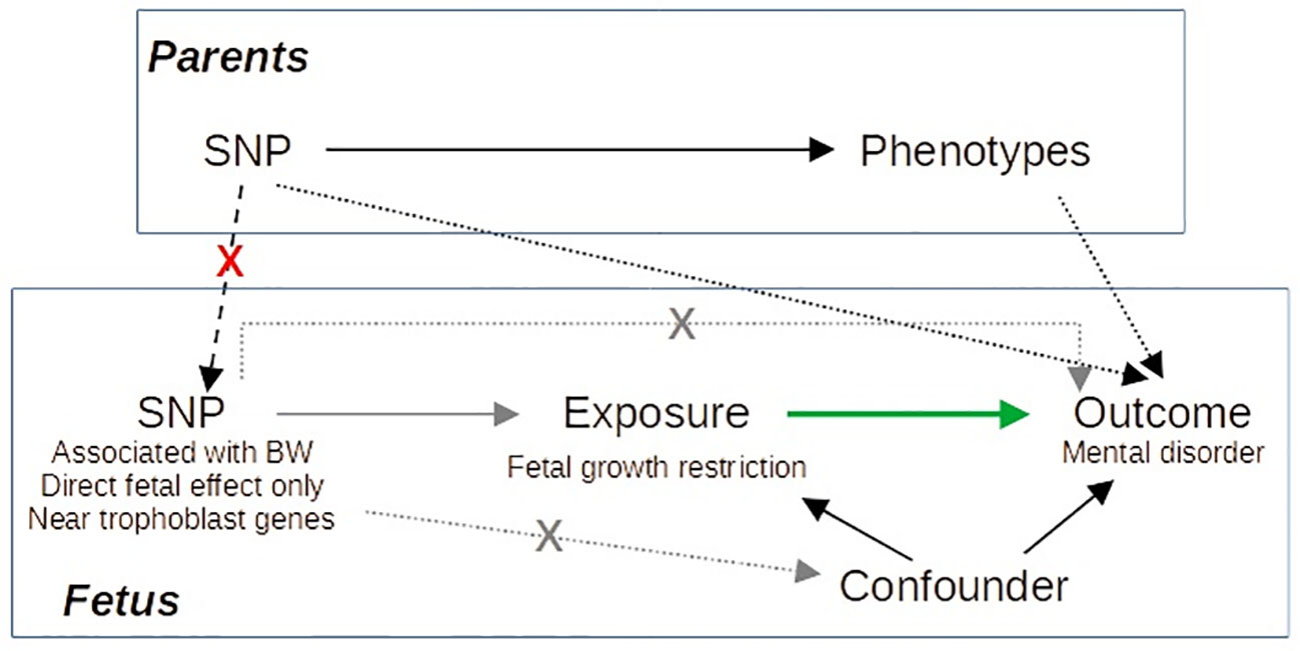
Directed acyclic graph illustrating our Mendelian randomization approach. Gray arrows correspond to the IV assumptions. Green arrow indicates the causal effect of interest. By selecting as IVs SNPs with specific characteristics, we avoid dynastic effects (dashed black arrows), as it is improbable that SNPs accomplishing these characteristics act on the outcome via parents’ phenotype.

Here, we perform an MR study of birth weight on mental disorders based on a larger GWAS than previous studies (23). Furthermore, we selected as instrumental variables (IVs) those SNPs with fetal effect and close to genes involved in trophoblast biology based on single-cell RNA sequencing studies of the decidual–placental interface (24, 25) to distinguish fetal adversity from constitutionally small newborns. By selecting as IVs, SNPs related to trophoblast biology with only direct fetal effect on birth weight, we limit the probability of dynastic effects, that is, the effect of the SNP on the offspring outcome via parental phenotype (**Figure 1**).

## 2 Materials and methods

**Figure 2:**
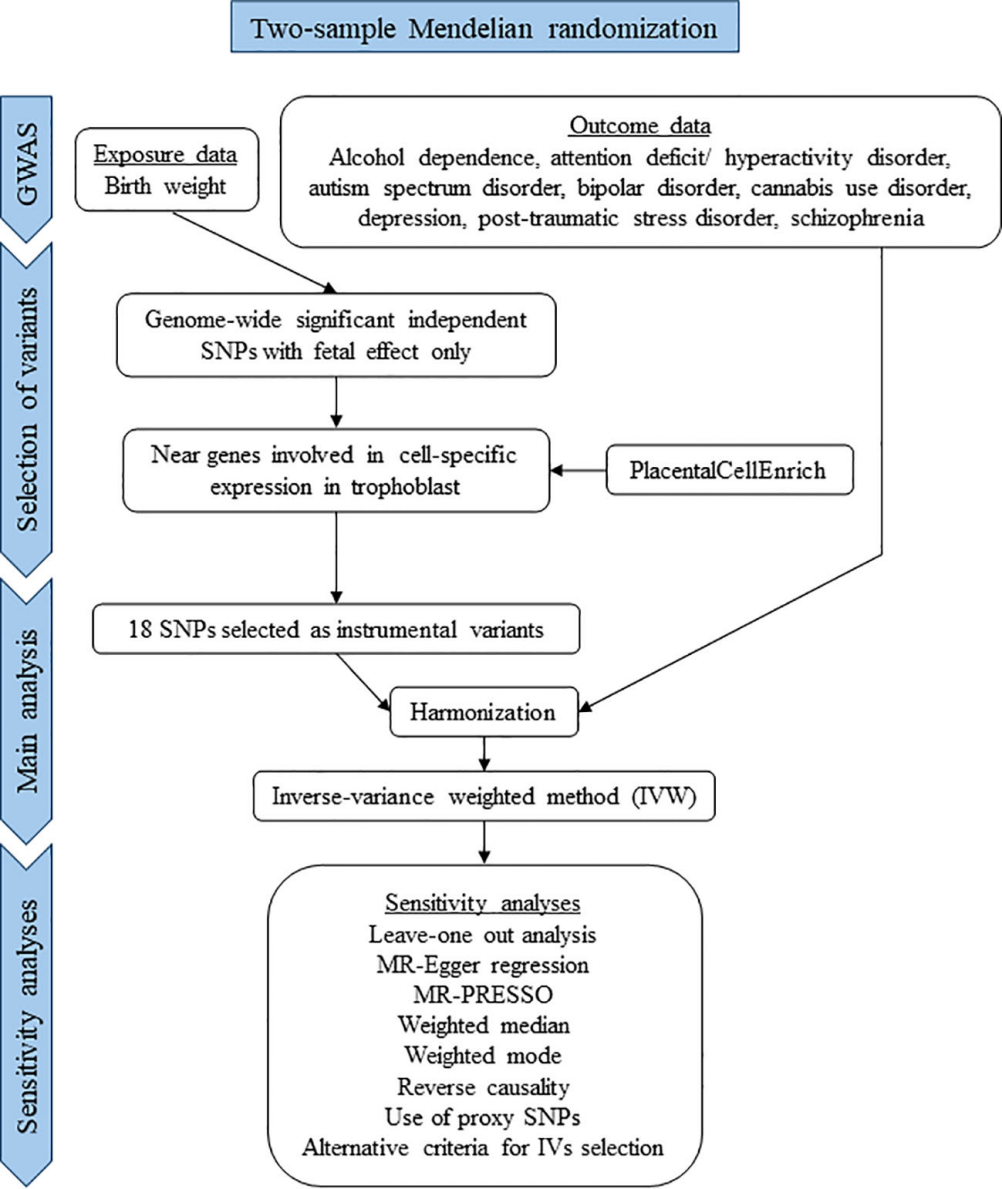
Flow chart of the study.

This study was conducted in accordance with the STROBE-MR (Strengthening the Reporting of Observational Studies in Epidemiology–Mendelian Randomization) guidelines for reporting MR studies (**Supplementary Table S1**) (26). A flow chart of the study is presented in **Figure 2**.

### 2.1 Selection of genetic instrumental variables

For an SNP to be a valid IV, it must meet the following conditions (referred to as core MR assumptions): it should be associated with the exposure (relevance assumption), not associated with confounders (independence assumption), and only related to the outcome through the exposure (exclusion restriction assumption) (27). SNPs used as IVs were taken from the currently largest GWAS meta-analysis on birth weight (*N* = 423,683, including samples from EGGC, UK Biobank, and the Icelandic birth register). Birth weight was normalized to a standard normal distribution using rank-based inverse normal transformation prior to analysis (23). The study included information on whether the variants exert their effect directly through the fetal genome or indirectly through the maternal genome, based on a subset of 104,920 Icelandic parent–offspring trios. The selected SNPs accomplished these conditions: (i) *P*< 5 × 10^−8^ in the main GWAS of birth weight, that is, genome-wide significant (GWS) SNPs, (ii) classified as acting exclusively through the fetal effect, and (iii) located near genes involved in trophoblast biology. Specifically, all annotated coding genes in the 300 kb region centered on each SNP were collected using the MAGMA (28) auxiliary file for the GRCh38 version and the R package “GenomicRanges 1.52.1” (29). Then, the PlacentalCellEnrich tool (30) was used to test for cell-specific expression of these genes in any of the three types of trophoblast cells identified in single-cell RNA sequencing of first-trimester human placenta, that is, villous cytotrophoblast, syncitiotrophoblast, and extravillous trophoblast (24, 25). All types of cell-specific expression defined by PlacentaCellEnrich were considered, corresponding to at least fivefold higher expression level in a particular cell type or group of cell types compared to all other cells or to average levels in all other cells. The default expression threshold of 1 was used. PlacentalCellEnrich was also used to test for cell-specific gene enrichment of the subset of genes around the GWS SNPs with fetal effects, using the hypergeometric test.

Additional IVs were selected in sensitivity analyses. Specifically, all human genes with direct or indirect relationships with the Gene Ontology (GO) (31, 32) term GO:0001892, embryonic placenta development, were retrieved using AmiGO 2 (33). SNPs were selected if present a *P*< 5 × 10^−8^ in the GWAS of birth weight and are at less than 150 kb of any gene from this list. Another group of SNPs selected as IVs were all independent GWS SNPs with predicted fetal effect by Juliusdottir et al. (23). Finally, subsets of SNPs were selected based on the trophoblast cell type where specific gene expression was detected.

To confirm the relevance of the SNPs selected as IVs in relation to trophoblast physiology, information on the chromatin state in E005 H1 BMP4 derived trophoblast cultured cells from the Roadmap Epigenomics Consortium (34) were retrieved using the WashU EpiGenome Browser (https://epigenomegateway.wustl.edu/). Specifically, SNPs were classified as located in active or inactive chromatin regions based on the 18-stated model.

As a measure of the strength of the association between each IV or the overall IV and the exposure, the *F*-statistic was computed for each SNP using the formula


$$F=\frac{R^{2}\;(N-k-1)}{1-R^{2}}$$

where *R* ^2^ is the proportion of variance explained by each SNP (or their sum, in case of overall IV), *N* is the sample size and *k* is equal to 1 when applied to each SNP and equal to the number of IVs when applied to the overall IV. *R* ^2^ was estimated as $2\beta^{2}MAF(1-MAF)$ , where β is the beta coefficient and MAF is the minor allele frequency.

### 2.2 Summary data for outcomes

Summary statistics from the largest available GWAS for eight psychiatric disorders with at least 10,000 cases have been selected for this study. The traits included were alcohol dependence (35), ADHD (36), ASD (37), BIP (38), cannabis use disorder (CUD) (39), depression (40), PTSD (41), and SCZ (42) (**Supplementary Table S2** lists all GWAS summary statistics used with descriptive information). All included GWAS are from European ancestry samples to ensure a similar pattern of linkage disequilibrium. As a sensitivity analysis of the main results, a GWAS of clinically ascertained depression from the Psychiatric Genomics Consortium (PGC), comprising 25% of the total depression cases, was used (43).

### 2.3 Mendelian randomization analysis

Two-sample MR analyses were performed between birth weight as exposure and all psychiatric outcomes using the R package “TwoSampleMR 0.5.6” (44). Prior to MR analyses, the exposure and outcome summary statistics were harmonized to ensure that the effects were referenced to the same allele. Palindromic SNPs with minor allele frequency (>0.4) were removed. In case of any IV not present in a GWAS, a search for proxy SNPs at *r* ^2^ > 0.8 with the IV was performed in the “LDlink” web service (45), using the “LDproxy” tool, genome version GRCh38, the 1000 Genomes Project European populations as reference, and a base pair window of ±150,000. As most MR methods are based on uncorrelated variants, TwoSampleMR includes a clumping step. This clumping step was performed with default parameters, removing the less significant SNP in the exposure GWAS at each pair with LD *r* ^2^ > 0.001 using the 1000 Genomes Project European populations as reference.

Summary statistics of alcohol dependence did not report effect size. In this case, the effect size was estimated from Z score using the following formula


$$\beta =\frac{z}{\sqrt{2p(1-p)\;(n+z^{2})}}$$

where β is the beta coefficient, z is the Z score, n is the sample size, and p is the effect allele frequency (46). This frequency was taken from the 1000 Genomes Phase 3 European reference panel. In order to estimate the standard errors of beta in ADHD, ASD, PTSD, and alcohol dependence summary statistics, the following formula was used:


$$SE=\frac{\beta }{z}$$

where *SE* is the standard error of the beta coefficient, β is the beta coefficient and *z* is the *Z* score (46).

The causal estimate for each SNP was measured as the ratio between the effect of the SNP on the outcome and the effect of the SNP on the exposure, that is, the Wald ratio estimate. Wald ratio estimates were meta-analyzed using the random effects inverse-variance weighted method (IVW) as the main MR method to obtain exposure effect size estimates. This is the more commonly used method and is the most powerful method in the absence of pleiotropy or if the pleiotropy is balanced (47, 48). Heterogeneity was tested using the Q-statistic. Significance for the main results was established as *P*< 0.00625, corresponding to a Bonferroni’s correction for test on eight outcomes. Considering the existence of genetic correlation between the different outcomes (49), this correction is conservative.

In case of a significant association detected by IVW, several sensitivity tests have been carried out to check the robustness of the results (9, 48). Leave-one-out analysis was performed to assess the effect of any single IV on the MR estimate. The MR-Egger regression (50) was used to allow for unbalanced pleiotropic effects. Egger-intercept was used to test for heterogeneity of Wald ratio estimates. MR-PRESSO was used to detect outliers. After outlier removal, MR-PRESSO performs an IVW approach (51). Two methods robust to outliers were used, the weighted median and the weighted mode. The first method is robust to up to 50% of SNPs violating the IV assumptions. The weighted mode is valid under the assumption that the largest subset of IVs with the same estimate are valid instruments, that is, the majority assumption. This method is sensitive to the bandwidth parameter that defines the clustering of IVs. The default value of 1 was used as first option, while other values were used to analyze the robustness of the method. Putative bias due to sample overlap between the exposure and outcome GWAS was quantified using the method proposed by Burgess and Davies (52). Reverse causality was tested using each psychiatric disorder as exposure and birth weight as outcome.

## 3 Results

### 3.1 Selection of SNPs as instrumental variables based on trophoblast-specific gene expression

There were 351 genes around the 87 lead GWS SNPs, including secondary signals, for fetal growth GWAS with predicted fetal effect. As expected, the most significantly enriched cell types detected by PlacentaCellEnrich corresponded to trophoblast (**Supplementary Figure S1**). Specifically, syncytiotrophoblasts were significant in the Suryawanshi et al.s’ dataset (24) (adjusted *P* = 0.047, fold-change = 2.97) and extravillous trophoblasts were significant in the Vento-Tormo et al.’s dataset (25) (adjusted *P* = 0.0035, fold-change = 4.82). There were 18 SNPs with a gene specifically expressed in trophoblast at less than 150 kb. These SNPs were selected as IVs (**Table 1**). Thirteen of the 18 SNPs were located in active chromatin regions of the E005 H1 BMP4–derived trophoblast cultured cells, while 80% of the average epigenome corresponds to inactive chromatin states (34).

**Table 1:** Instrumental variables (IVs) selected for the Mendelian randomization (MR) analysis.

SNP ID^1^	Position hg38	Alleles^2^	EAF^3^	Beta (s.e.) birth weight	P birth weight	Trophoblast gene (distance)	Trophoblast cell type^4^	Nearest gene (distance)	*R* ^2^	*F*-statistic
**rs12401656**	Chr1:42991096	A/G	0.1272	−0.0277(0.0036)	2.4E-14	*SLC2A1*(32228)	SCT	*SLC2A1*(32228)	1.78E-04	75.51
**rs141845046**	Chr1:155015228	T/C	0.0298	0.0746(0.008)	1.6E-20	*EFNA1*(112648)	EVT, SCT	*ZBTB7B*(0)	2.89E-04	122.67
rs10913200	Chr1:176552519	A/G	0.0229	−0.0552(0.0078)	1.4E-12	*PAPPA2*(0)	EVT	*PAPPA2*(0)	1.48E-04	62.53
**rs12656216**	Chr5:36160566	G/A	0.2247	−0.0196(0.003)	7.4E-11	*SKP2*(0)	EVT, VCT	*SKP2*(0)	1.29E-04	54.56
**rs75104038**	Chr6:34222327	A/G	0.0447	0.0465(0.0053)	2.9E-18	*HMGA1*(14546)	VCT	*HMGA1*(14546)	2.20E-04	93.42
**rs7771453**	Chr6:35530855	G/A	0.2097	0.0243(0.003)	3.9E-16	*TEAD3*(33776)	SCT	*TULP1*(17986)	2.05E-04	87.05
**rs577204588**	Chr6:53156939	C/T	0.0010	−0.1535(0.0234)	5.8E-11	*GCM1*(8098)	EVT, SCT	*GCM1*(8098)	2.48E-04	105.09
**rs34776209**	Chr7:23473474	T/C	0.2386	−0.0239(0.0029)	6.9E-17	*IGF2BP3*(2983)	VCT	*IGF2BP3*(2983)	2.07E-04	87.67
**rs1323438**	Chr9:116353252	T/C	0.2724	−0.0203(0.0027)	1.3E-13	*PAPPA*(0)	EVT	*PAPPA*(0)	1.70E-04	72.06
rs2901307	Chr10:122368927	C/T	0.5328	−0.0213(0.0025)	5.6E-18	*TACC2*(114385)	SCT	*PLEKHA1*(5781)	2.27E-04	96.04
rs1011476	Chr11:2277805	T/G	0.2465	0.0179(0.0027)	4E-11	*ASCL2*(7217)	EVT	*ASCL2*(7217)	1.30E-04	55.15
**rs234864**	Chr11:2836067	G/A	0.4523	−0.0169(0.0025)	1.2E-11	*CDKN1C*(47146), *PHLDA2*(92206)	EVT, SCT | EVT, SCT, VCT	*KCNQ1*(0)	1.42E-04	60.22
rs4444073	Chr11:10310114	C/A	0.4732	−0.0219(0.0024)	2.8E-19	*ADM*(2722)	EVT	*ADM*(2722)	2.39E-04	101.39
rs12584892	Chr13:73050396	T/C	0.1710	−0.0196(0.0032)	1.3E-09	*KLF5*(4580)	VCT	*KLF5*(4580)	1.14E-04	48.30
**rs222857**	Chr17:7261244	C/T	0.4205	−0.0282(0.0025)	2.2E-30	*CLDN7*(0)	EVT, SCT, VCT	*CLDN7*(0)	3.90E-04	165.37
**rs41355649**	Chr19:33299650	A/G	0.0646	−0.0366(0.0051)	5E-13	*CEBPA*(284)	SCT	*CEBPA*(284)	1.63E-04	69.09
**rs753381**	Chr20:41168825	T/C	0.4602	0.0154(0.0024)	2.8E-10	*TOP1*(44338)	SCT	*PLCG1*(0)	1.18E-04	49.89
**rs220193**	Chr21:42161198	A/G	0.2346	0.021(0.003)	1.5E-12	*ABCG1*(38491)	SCT	*UMODL1(17745)*	1.53E-04	64.76

^1^SNPs located in active chromatin states in E005 H1 BMP4 derived trophoblast cultured cells from the Roadmap Epigenomics Consortium are in boldface.

^2^First allele is the effect allele.

^3^Effect allele frequency in the 1000 Genomes reference panel (European population).

^4^EVT, extravillous trophoblasts; SCT, syncytiotrophoblasts; VCT, villous cytotrophoblasts.

Individual *F*-statistic ranged from 48.30 to 165.37, indicative of no problems with weak instrument bias. The total number of IVs common to each exposure-outcome analysis was 13–15. The overall *F*-statistic ranged from 78.21 (depression) to 84.18 (ADHD). The mean *F*-statistic ranged from 78.63 (depression) to 83.99 (ADHD). The rest of the outcomes employ the same 15 IVs whose overall *F*-statistic value is 81.78 and mean *F*-statistic is 81.56. Of the selected IVs (**Table 1**), rs5777204588 and its eight proxy SNPs were not found in any of the summary statistics of the outcomes. rs141845046 was not found in the GWAS of depression, and rs10913200 and rs41355649 were not found in the GWAS of ADHD. There was a proxy SNP, rs73024215, for one of these SNPs, rs41355649 (*r* ^2^ = 0.87), but it was absent from the ADHD GWAS. As for the SNPs rs7771453 and rs1011476, they were removed in the clumping step in all MR analyses.

### 3.2 Main Mendelian randomization analyses

**Figure 3:**
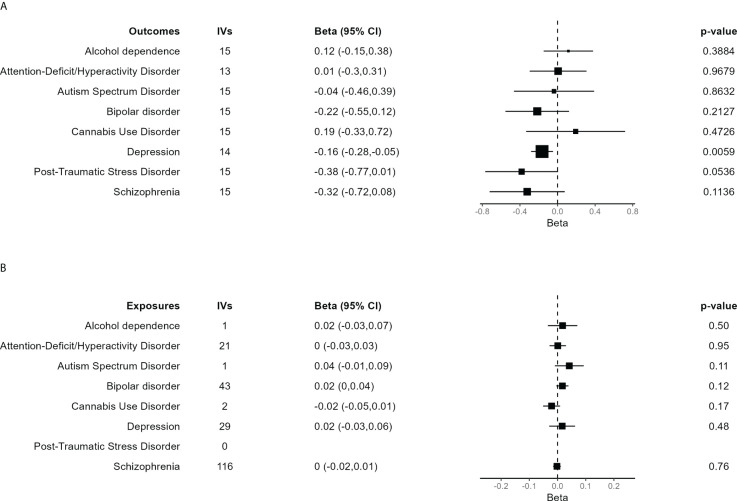
Forest plot of the main results of the Mendelian randomization (MR) analysis. **(A)** Results of the MR analysis using the different psychiatric disorders as outcome. The size of each square is proportional to the effective sample size of each GWAS (**Supplementary Table S2**). **(B)** Results of the MR analysis in which independent SNPs significantly associated with each of the psychiatric disorders were taken as IVs to assess their effect on birth weight, evaluating the inverse causality hypothesis. In case of just one IV, the Wald ratio test is shown. The other results are based on the IVW method.

Main results for the MR analyses for each outcome are shown in **Figure 3A**. IVW method based on 14 trophoblast IVs was significant for depression after correction for multiple tests (β = −0.165, 95% CI = −0.282 to −0.047, *P* = 0.0059). The association is in the expected direction, that is, lower birth weighted is causally associated with increased risk of depression. No other exposure outcome was significant (*P* > 0.05 in all cases). When birth weight was considered the outcome and each psychiatric disorder the exposure, there were no association, although PTSD was not tested due to lack of IVs (**Figure 3B**).

Assuming that the 217,397 samples from UKBB at the birth weight GWAS are also among the 361,315 samples from UKBB at the depression GWAS, and that the 11,526 samples from Iceland at the depression GWAS are also among the 125,541 samples from Iceland at the birth weight GWAS, the maximum sample overlap is around 45% of the depression samples. Taking into the lower limit of the *F*-statistic 95% CI, 71.15, the estimated bias associated with overlap was negligible.

### 3.3 Sensitivity analysis for the birth weight–depression association

**Figure 4:**
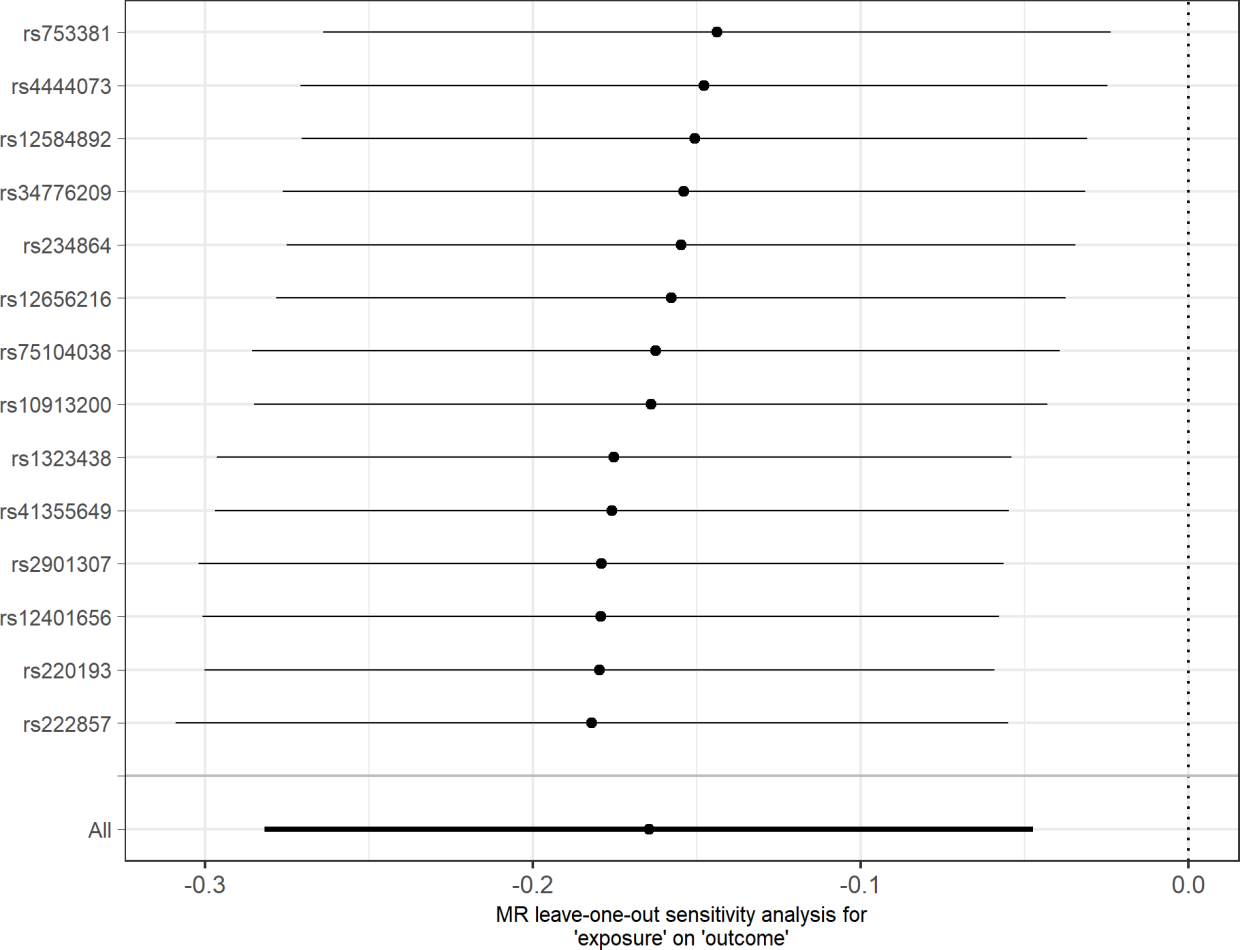
Leave-one-out sensitivity analysis for depression as outcome. The *y*-axis indicates the SNP that is removed in each analysis. The *x*-axis shows the beta (dot) and 95% CI (line) for each analysis.

**Figure 5:**
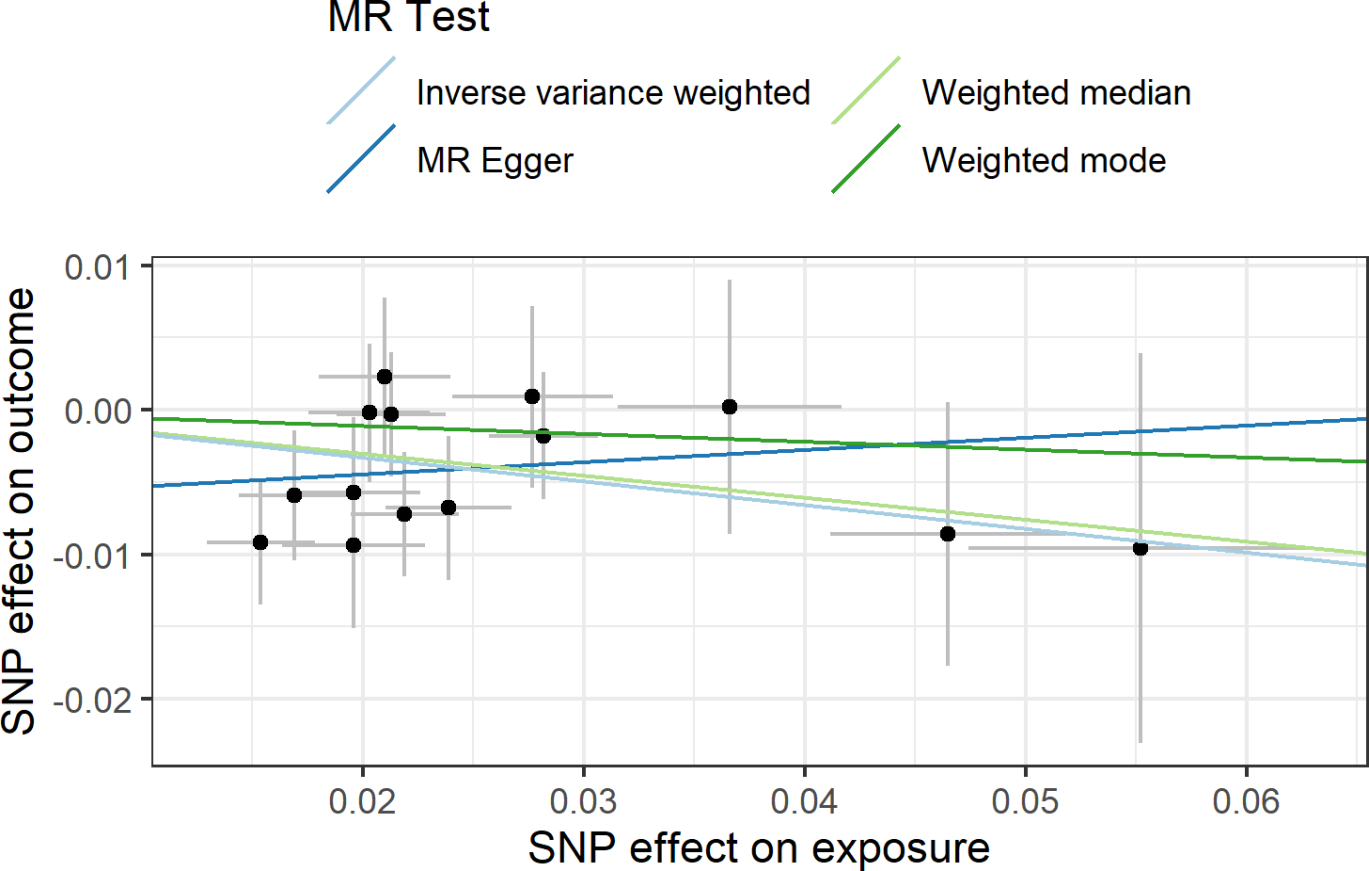
Scatter plot showing the beta effects of SNPs on exposure (birthweight) and outcome (depression). The estimates are represented as dots, with 95% CI represented by horizontal and vertical lines, respectively. The slope of each colored line corresponds to the estimated causal effect by each MR method.

In agreement with the absence of heterogeneity (*Q* = 9.07, *P* = 0.77), the association remained significant in leave-one-out analysis (**Figure 4**). Furthermore, MR-PRESSO did not detect any outlier using the default outlier significance threshold of 0.05. Egger intercept was not significant (intercept = −0.006, 95% CI = −0.004 to −0.016, *P* = 0.24), suggesting absence of directional (or unbalanced) pleiotropy. The MR-Egger estimate was not significant (β = 0.084, 95% CI = −0.324 to 0.493, *P* = 0.69), and the causal estimate is in the opposite direction (**Figure 5**). However, there is absence of heterogeneity (*Q*’ = 7.514, *P* = 0.82), and the difference *Q* – *Q*’ was not significant (1.556, *P* = 0.21), indicating that MR-Egger does not fit substantially better to the data. The weighted median estimate was similar to the IVW (β = −0.152, 95% CI = −0.313 to 0.009) (**Figure 5**). The result is near significance (*P* = 0.0638), in agreement with the low power of the method. Finally, the weighted mode estimate using the default bandwidth of 1 was lower and insignificant (β = −0.054, 95% CI = −0.285 to 0.176, *P* = 0.65) (**Figure 5**). However, the use of different bandwidths gave rise to different conclusions. For instance, the causal estimate using a bandwidth parameter of 2 is more similar to that of the IVW method (β = −0.13, 95% CI = −0.28 to −0.02, *P* = 0.089). Using a bandwidth of 2.5 the MR estimate reached significance (β = −0.139, 95% CI = −0.279 to 0.000, *P* = 0.050).

In order to test the effect of different definitions of depression on the results, we analyzed the subsample of depression comprising only clinically ascertained samples from the PGC. In this sample, the IVW analysis was not significant (β = −0.087, 95% CI = −0.340 to 0.166, *P* = 0.498).

**Figure 6:**
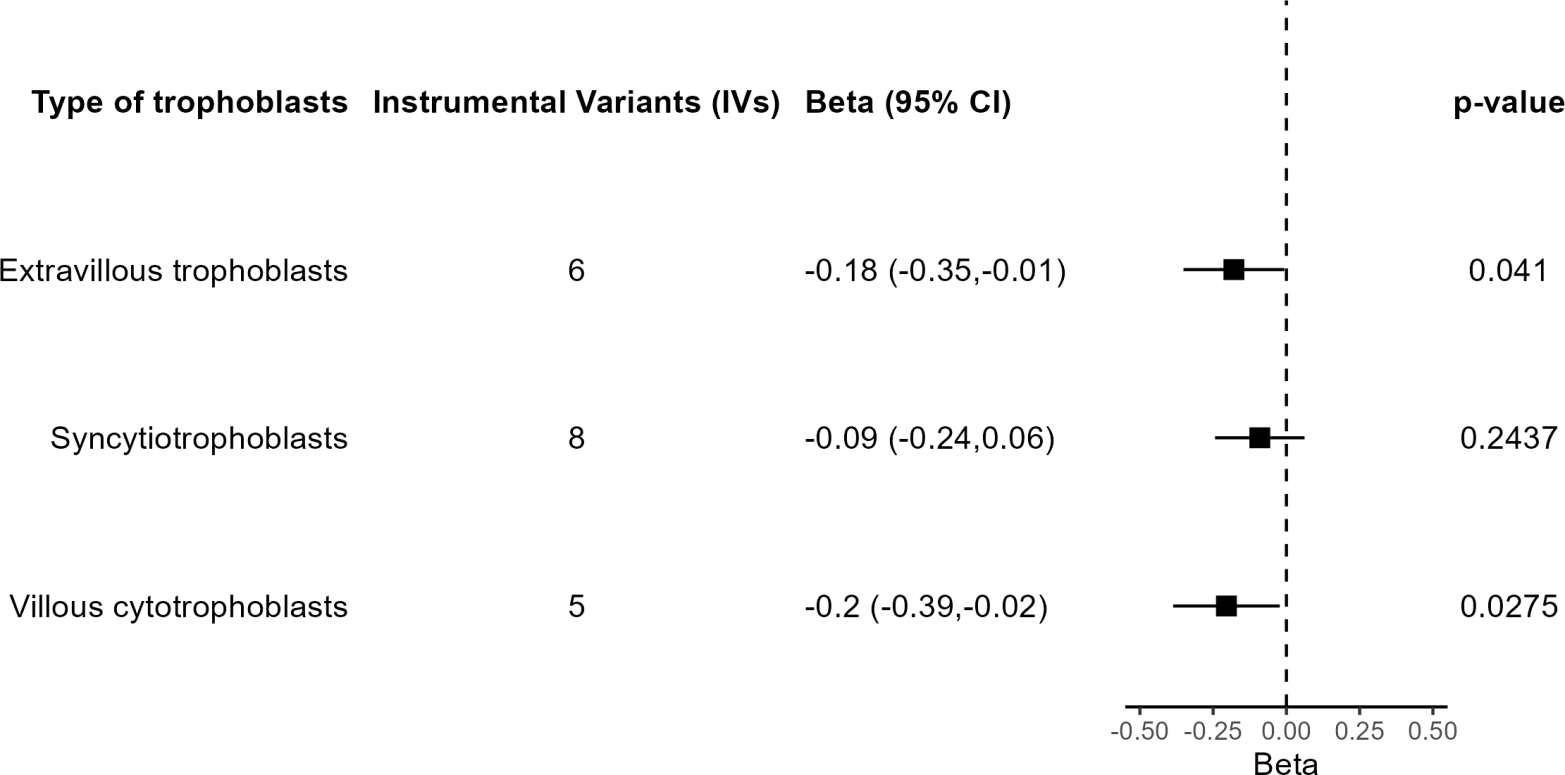
Results of the MR analysis based on subsets of IVs according to trophoblast cell type.

A final set of sensitivity analyses considered distinct IV selection. To test for the effect of trophoblast specific expression, MR IVW was performed using all independent GWS SNPs for birth weight with predicted fetal effect, *n* = 79. The causal effect estimate was not significantly different from 0 (β = −0.009, 95% CI = −0.092 to 0.075, *P* = 0.84), in agreement with a specific role for trophoblast physiology. Another IV selection included 11 SNPs located near genes involved in embryonic placental development according to GO (**Supplementary Table S3**). Five SNPs are common to those from the main analysis based on trophoblast specific expression. Once again, the result lacked significance (β = −0.019, 95% CI = −0.175 to 0.136, *P* = 0.81). Last, when the IVs were selected based on the specific trophoblast cell type where specific gene expression was present, IVW estimates were significant in extravillous trophoblasts and villous cytotrophoblasts but not in syncytiotrophoblasts (**Figure 6**).

## 4 Discussion

Using an MR approach, we present evidence for the involvement of placenta in susceptibility to broadly defined depression. Specifically, we selected SNPs with fetal effect on birth weight and near genes with differential expression in trophoblasts as IVs for oxygen and nutrient supply to the fetus. However, we did not detect any significant result with other psychiatric or substance use disorders. Reverse causality was also discarded, as none of the sets of IVs chosen for each disorder had a significant effect on birth weight.

A previous MR study analyzed the effects of birth weight on several psychiatric disorders, using a different approach, as their SNP effects were direct fetal effects adjusted for maternal genotype (12). In contrast with our results, the authors found evidence of an effect of birth weight on ADHD and PTSD but not on depression. These different findings may also be related to the selection of IVs. We have chosen SNPs with fetal effect on birth weight and near genes with differential gene expression in trophoblasts as a way to identify IVs specifically involved in oxygen and nutrient supply to the fetus instead of SNPs related to normal growth in constitutionally small but healthy newborns. The relevance of the selection based on differential gene expression in trophoblast was confirmed by lack of significance in sensitivity analyses in depression using as IVs all SNPs with fetal effect on birth weight or SNPs near genes within the GO term “embryonic placenta development.” The use of larger exposure and outcome GWAs in our study may also contribute to differences with the previous one (12).

The outcome GWAS used for depression was based on a highly heterogeneous phenotype, which includes both people with self-reported past help seeking for problems with “nerves, anxiety, tension or depression” (termed “broad depression”) as well as patients ascertained with a diagnostic interview from the PGC, representing about 75% and 25% of the total sample, respectively (40). In contrast to the main result based on the total samples, the sensitivity analysis including only the clinically ascertained samples was not significant. This may be interpreted considering that the genetic susceptibility associated with minimal phenotyping definitions of depression is less specific to depression, probably incorporating genetic susceptibility to different psychiatric disorders (53, 54). Thus, it may be possible that the causal association we detected was the only significant result in MR due to higher power instead of being specific to depression. This agrees with the epidemiological study of Pettersson et al. (8), who found an association of birth weight with a general psychopathology factor. Alternatively, GWAS associated to broad depression may identify different type of variants, such as those related to personality traits and disorders or to nonspecific subclinical depressive symptoms secondary to other traits (53, 54).

The causal effect of trophoblast functioning on susceptibility to psychopathology may be related to the process of invasion of the uterine walls that takes place in the early stages of placentation. The aim of this process is the remodeling of the uterine arteries to provide the fetus a sufficient and permanent supply of oxygen and nutrient (15, 16). A problem in the migration capacity of the trophoblasts could potentially result in a reduced number of invaded blood vessels leading to an incomplete remodeling process of the uterine arteries. The reduced blood flow to the fetus will cause a lower or intermittent provision of nutrients and oxygen, and there could be episodes of oxidative stress that can lead to a misfolded protein accumulation (55, 56). Hypoxic conditions have also been linked to a reduced activity of differentiation pathways of trophoblasts, such as syncytialization (57, 58). Thus, a maternal–fetal interface imbalance mediated by impaired trophoblast function may compromise the normal development of the large human fetal brain, considering its high demand of oxygen and nutrients (15, 59). Trophoblast invasiveness has recently been suggested as a candidate mechanism to explain the interaction of PlacPRS and obstetric complications on early neurodevelopmental impairment (60).

## 5 Limitations

The findings in this work must be interpreted in the context of an MR approach. The reliability of MR results is based on several IV assumptions. While the strength of the association between IVs and exposure was confirmed by the *F*-statistics, other assumptions cannot be formally tested (9). Several sensitivity analyses, such use of methods robust to outliers or analysis of heterogeneity in IVs effects were used to deal with putative horizontal pleiotropy. In addition, we selected IVs near genes with differential expression in trophoblast, as this is the cell type with a more important role in establishment and maintenance of a maternal–fetal interface. Several of the selected genes, such as *ADM*, *ASCL2*, *CDKN1C*, *GCM1*, *HMGA1*, *IGF2BP3*, *PAPPA*, *PAPPA2*, and *PHLDA2*, play a known role in trophoblast physiology and related pathologies, such as intrauterine growth restriction (IUGR) or preeclampsia (61–68). However, we cannot discard an effect of the selected IVs by another mechanism not related with fetal growth restriction.

Another limitation was the low sample size of some of the GWAS of psychiatric disorders reducing the power of the analysis. This is especially problematic in case of substance use disorders. This low power may also be the reason for non-significance of the weighted median analysis in spite of similar effect than the IVW method. Increase of knowledge of trophoblast physiology in near future may lead to identification of more IVs as a way to increase power in addition to increase sample size in GWAS. Finally, the studies included in the PlacentalCellEnrich database (30) use expression data for placentas in the first three months of pregnancy. Although this period is key for establishment of the maternal–fetal interface, it would be necessary to include results from placentas in the second and third trimester to obtain a more complete view of placenta-associated variants.

## 6 Conclusion

In summary, we have identified a causal effect of placental impairment in broadly defined depression, probably reflecting an unspecific psychopathology. This adds new data on the current debate about placental role in mental health (21, 60, 69, 70). Although no treatments for fetal growth restriction are currently available, there are several promising agents on clinical trials or preclinical research, acting on processes such as oxygen supply by angiogenesis or vasodilatation, or mitigation of oxidative stress (71, 72). Therefore, further research is needed to confirm this causal effect, as it could have clear implications in prevention of mental disorders in near future.

## Data availability statement

Publicly available datasets were analyzed in this study. This data can be found here: https://www.decode.com/summarydata/ https://pgc.unc.edu/for-researchers/download-results/.

## Ethics statement

All the data used in this work are de-identified summary statistics data made publicly available after approval by the respective institutional ethical committees of the different consortia. Therefore, no new ethical approval or consent was required.

## Author contributions

PJ-F: Data curation, Formal analysis, Investigation, Methodology, Writing – original draft, Writing – review & editing. JC: Conceptualization, Funding acquisition, Investigation, Resources, Supervision, Validation, Writing – original draft, Writing – review & editing.
