# Supplementary material for: Exploring the causal effect of placental physiology in susceptibility to mental and addictive disorders: a Mendelian randomization study
Source: Front Psychiatry. 2024 Jul 29;15:1396837. doi: 10.3389/fpsyt.2024.1396837 (PMC11317394; doi:10.3389/fpsyt.2024.1396837)
Supplement: Supplementary file 1 [file DataSheet_1.pdf]

Supplementary Material

1 Supplementary Figures and Tables

1.1 Supplementary Figures

Supplementary Figure 1. Most significantly enriched cell types detected by each dataset in PlacentaCellEnrich (1). Vento-Tormo dataset (2) (**A**) and Suryawanshi dataset (3) (**B**).

A

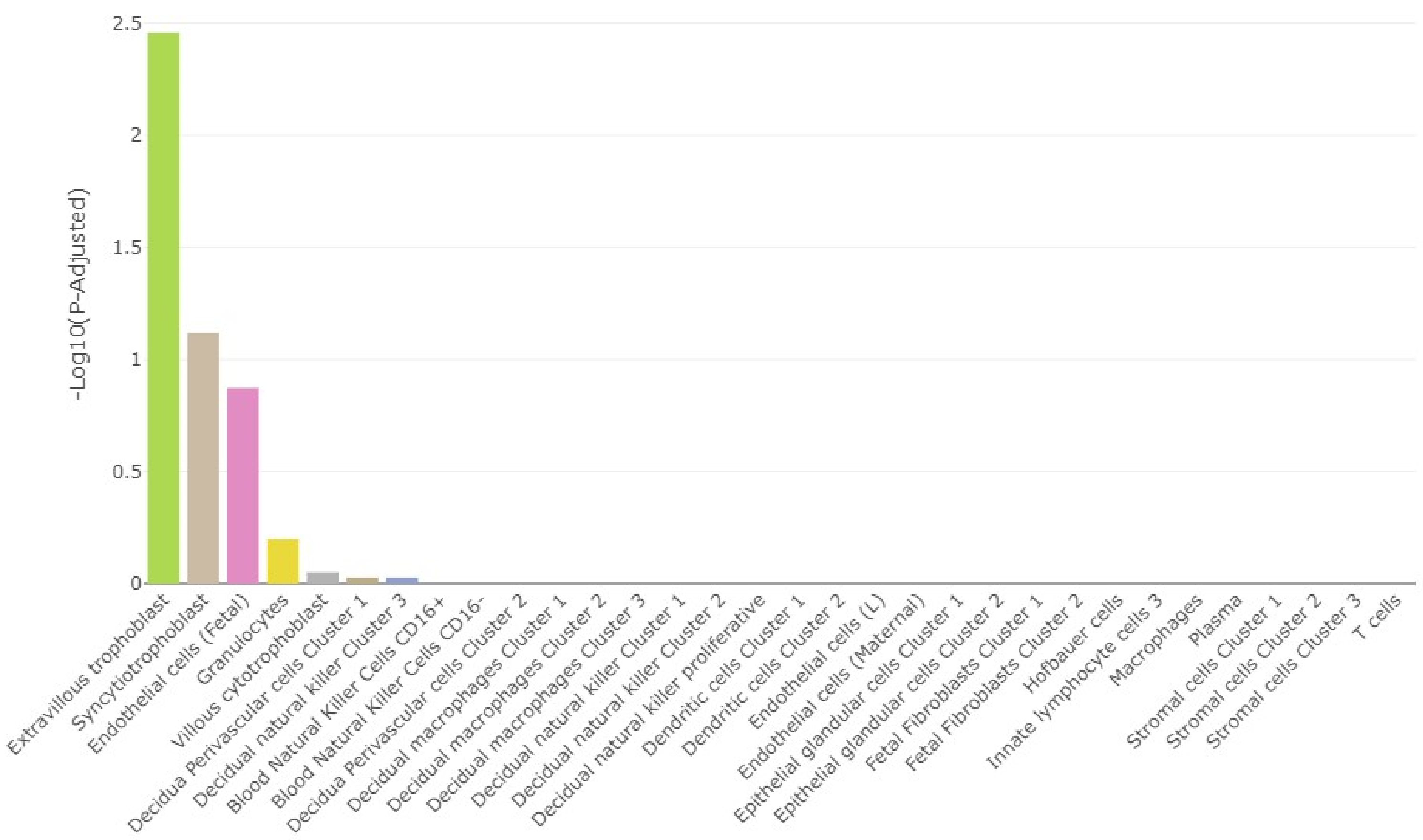

-Log10(P-Adjusted)
2.5
2
1.5
1
0.5
0
Extravillous trophoblast
Syncytiotrophoblast
Endothelial cells (Fetal)
Granulocytes
Villous cytotrophoblast
Decidua Perivascular cells Cluster 1
Decidual natural killer Cluster 3
Blood Natural Killer Cells CD16+
Blood Natural Killer Cells CD16-
Decidua Perivascular cells Cluster 2
Decidual macrophages Cluster 1
Decidual macrophages Cluster 2
Decidual macrophages Cluster 3
Decidual natural killer Cluster 1
Decidual natural killer Cluster 2
Decidual natural killer proliferative
Dendritic cells Cluster 1
Dendritic cells Cluster 2
Endothelial cells (L)
Endothelial cells (Maternal)
Epithelial glandular cells Cluster 1
Epithelial glandular cells Cluster 2
Fetal Fibroblasts Cluster 1
Fetal Fibroblasts Cluster 2
Hofbauer cells
Innate lymphocyte cells 3
Macrophages
Plasma
Stromal cells Cluster 1
Stromal cells Cluster 2
Stromal cells Cluster 3
T cells

B

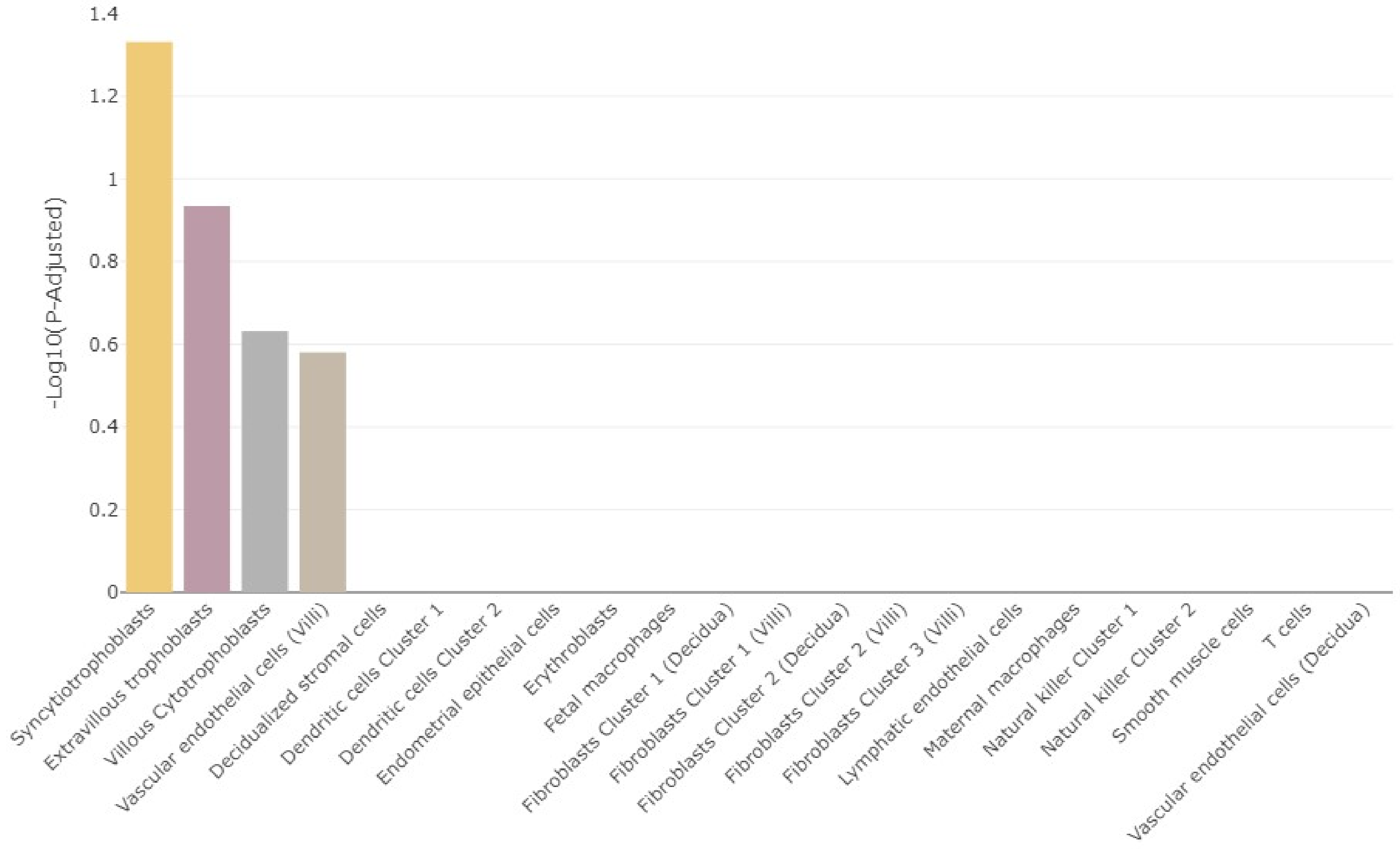

1.4
1.2
1
0.8
0.6
0.4
0.2
0
-Log10(P-Adjusted)
Syncytiotrophoblasts
Extravillous trophoblasts
Villous Cytotrophoblasts
Vascular endothelial cells (Villi)
Decidualized stromal cells
Dendritic cells Cluster 1
Dendritic cells Cluster 2
Endometrial epithelial cells
Erythroblasts
Fetal macrophages
Fibroblasts Cluster 1 (Decidua)
Fibroblasts Cluster 1 (Villi)
Fibroblasts Cluster 2 (Decidua)
Fibroblasts Cluster 2 (Villi)
Fibroblasts Cluster 3 (Villi)
Lymphatic endothelial cells
Maternal macrophages
Natural killer Cluster 1
Natural killer Cluster 2
Smooth muscle cells
T cells
Vascular endothelial cells (Decidua)

1.2 Supplementary Tables

Supplementary Table 1. STROBE-MR checklist (4).

Item No.	Section	Checklist item	Location / relevant text from manuscript
**TITLE AND ABSTRACT**			
1	Title and abstract	Indicate Mendelian randomization (MR) as the study's design in the title and/or the abstract if that is a main purpose of the study	Title: "Exploring the causal effect of placental physiology in susceptibility to mental and addictive disorders: a Mendelian randomization study"<br>Abstract: "We conducted a two-sample summary-data Mendelian randomization study using as instrumental variables genetic variants strongly associated with birth weight, whose effect is exerted through the fetal genome, and are located near genes with differential expression in trophoblasts."
**INTRODUCTION**			
2	**Background**	Explain the scientific background and rationale for the reported study. What is the exposure? Is a potential causal relationship between exposure and outcome plausible? Justify why MR is a helpful method to address the study question	Introduction, par. 1-3:<br>"Epidemiological data revealed an association between prenatal/perinatal problems, such as gestational diabetes, gestational hypertension, maternal infections during gestation, nutritional deficits during gestation, or preeclampsia and mental disorders (1–3). These agrees with..."
3	**Objectives**	State specific objectives clearly, including pre-specified causal hypotheses (if any). State that MR is a method that, under specific assumptions, intends to estimate causal effects	Introduction, par. 3:<br>"However, association does not imply causality. Mendelian randomization (MR) is a methodological approach to test for causality using genetic predisposition as a proxy for the exposure factor (9)."<br>Introduction, par. 6:<br>"Here, we perform a MR study of birth weight on mental disorders based on a larger GWAS than previous studies (23). Furthermore, we selected as instrumental variables (IVs) those SNPs with fetal effect and close to genes involved in trophoblast biology based on single-cell RNA sequencing studies of the decidual-placental

			interface (24,25) to distinguish fetal adversity from constitutionally small newborns. "
**METHODS**			
4	**Study design and data sources**	Present key elements of the study design early in the article. Consider including a table listing sources of data for all phases of the study. For each data source contributing to the analysis, describe the following:	
	a)	Setting: Describe the study design and the underlying population, if possible. Describe the setting, locations, and relevant dates, including periods of recruitment, exposure, follow-up, and data collection, when available.	Materials and Methods, subsection "Selection of genetic instrumental variables", par. 1:<br>"SNPs used as IVs were taken from the currently largest GWAS meta-analysis on birth weight (N = 423,683, including samples from EGGC, UK Biobank, and the Icelandic birth register)."<br>Materials and Methods, subsection "Summary data for outcomes":<br>"All included GWAS are from European ancestry samples to ensure similar pattern of linkage disequilibrium."<br>Supplementary Table 2.
	b)	Participants: Give the eligibility criteria, and the sources and methods of selection of participants. Report the sample size, and whether any power or sample size calculations were carried out prior to the main analysis	Supplementary Table 2.<br>Power calculations were not carried out, but we have used the largest GWAS to date and consider only those GWAS with large sample size.<br>Materials and Methods, subsection "Summary data for outcomes":<br>"Summary statistics from the largest available GWAS for eight psychiatric disorders with at least 10,000 cases have been selected for this study."
	c)	Describe measurement, quality control and selection of genetic variants	Materials and Methods, subsection "Selection of genetic instrumental variables", par. 1:<br>"The selected SNPs accomplished these conditions: i) P < 5x10-8 in the main GWAS of birth weight…"
	d)	For each exposure, outcome, and other relevant variables, describe methods of assessment and diagnostic	Supplementary Table 2.

		criteria for diseases	
	e)	Provide details of ethics committee approval and participant informed consent, if relevant	Materials and Methods, subsection “Ethical considerations”: “All the data used in this work are de-identified summary statistics data made publicly available after approval by the respective institutional ethical committees of the different consortia. Therefore, no new ethical approval or consent were required.”
5	**Assumptions**	Explicitly state the three core IV assumptions for the main analysis (relevance, independence and exclusion restriction) as well assumptions for any additional or sensitivity analysis	Materials and Methods, subsection “Selection of genetic instrumental variables”, par. 1: “For a SNP to be a valid IV, it must meet the following conditions (referred to as core MR assumptions): it should be associated with the exposure (relevance assumption), not associated with confounders (independence assumption) and only related to the outcome through the exposure (exclusion restriction assumption) (27).”
6	**Statistical methods: main analysis**	Describe statistical methods and statistics used	
	a)	Describe how quantitative variables were handled in the analyses (i.e., scale, units, model)	Materials and Methods, subsection “Selection of genetic instrumental variables”, par. 1: “Birth weight was normalized to a standard normal distribution using rank-based inverse normal transformation prior to analysis (23).”
	b)	Describe how genetic variants were handled in the analyses and, if applicable, how their weights were selected	Materials and Methods, subsection “Selection of genetic instrumental variables”, par. 1: “The selected SNPs accomplished these conditions: i) P < 5x10-8 in the main GWAS of birth weight …” Materials and Methods, subsection “Mendelian randomization analysis”, par. 1: “Prior to MR analyses, the exposure and outcome summary statistics were harmonized to ensure that the effects were referenced to the same allele. Palindromic SNPs with minor allele frequency (> 0.4) were removed…”
	c)	Describe the MR estimator (e.g., two-stage least squares, Wald ratio) and related statistics. Detail the included	Materials and Methods, subsection “Mendelian randomization analysis”, par. 3: “The causal estimate for each SNP was measured as the ratio

		covariates and, in case of two-sample MR, whether the same covariate set was used for adjustment in the two samples	between the effect of the SNP on the outcome and the effect of the SNP on the exposure, i.e., the Wald ratio estimate. Wald ratio estimates were meta-analyzed using the random effects inverse-variance weighted method (IVW) as the main MR method to obtain exposure effect size estimates.”
	d)	Explain how missing data were addressed	Materials and Methods, subsection “Mendelian randomization analysis”:<br>“In case of any IV not present in a GWAS, a search for proxy SNPs at $r^2 > 0.8$ with the IV was performed in the “LDlink” web service (44) …”
	e)	If applicable, indicate how multiple testing was addressed	Materials and Methods, subsection “Mendelian randomization analysis”, par. 3:<br>“Significance for the main results was established as $P < 0.00625$, corresponding to a Bonferroni’s correction for test on eight outcomes.”
7	**Assessment of assumptions**	Describe any methods or prior knowledge used to assess the assumptions or justify their validity	Materials and Methods, subsection “Selection of genetic instrumental variables”, par. 1:<br>“The selected SNPs accomplished these conditions: i) P < 5x10-8 in the main GWAS of birth weight […] Then, the PlacentalCellEnrich tool (30) was used to test for cell-specific expression of these genes in any of the three types of trophoblast cells identified in single-cell RNA sequencing of first trimester human placenta, i.e., villous cytotrophoblast, syncitiotrophoblast, and extravillous trophoblast (24,25).”<br>Materials and Methods, subsection “Selection of genetic instrumental variables”, par. 4:<br>“As a measure of the strength of the association between each IV or the overall IV and the exposure, the F-statistic was computed for each SNP using the formula…”
8	**Sensitivity analyses and additional analyses**	Describe any sensitivity analyses or additional analyses performed (e.g., comparison of effect estimates from different approaches, independent replication, bias analytic techniques,	Materials and Methods, subsection “Selection of genetic instrumental variables”, par. 2:<br>“Additional IVs were selected in sensitivity analyses. Specifically, all human genes with direct or indirect relationships with the Gene Ontology (GO) (31,32) term GO:0001892, embryonic placenta

		validation of instruments, simulations)	development, were retrieved using AmiGO 2 (33).”<br>Materials and Methods, subsection “Mendelian randomization analysis”, par. 4.<br>“In case of a significant association detected by IVW, several sensitivity tests have been carried out to check the robustness of the results (9,47) …”<br>Materials and Methods, subsection “Summary data for outcomes”:<br>“As a sensitivity analysis of the main results, a GWAS of clinically ascertained depression from the Psychiatric Genomics Consortium (PGC), comprising 25% of the total depression cases, was used (43).”
9	**Software and pre-registration**		
	a)	Name statistical software and package(s), including version and settings used	Materials and Methods, subsection “Mendelian randomization analysis”, par. 1:<br>“Two-sample MR analyses were performed between birth weight as exposure and all psychiatric outcomes using the R package "TwoSampleMR 0.5.6" (43)."
	b)	State whether the study protocol and details were pre-registered (as well as when and where)	The study protocol was not pre-registered.
**RESULTS**			
10	**Descriptive data**		
	a)	Report the numbers of individuals at each stage of included studies and reasons for exclusion. Consider use of a flow diagram	Not applicable to our study, as it is based on summary statistics from previous GWAS.
	b)	Report summary statistics for phenotypic exposure(s), outcome(s), and other relevant variables (e.g., means, SDs, proportions)	Supplementary Table 2.
	c)	If the data sources include meta-analyses of previous studies, provide the assessments of heterogeneity	Not applicable.

		across these studies	
	d)	For two-sample MR:<br>i. Provide justification of the similarity of the genetic variant-exposure associations between the exposure and outcome samples	Materials and Methods, subsection "Selection of genetic instrumental variables", par. 1:<br>"SNPs used as IVs were taken from the currently largest GWAS meta-analysis on birth weight (N = 423,683, including samples from EGGC, UK Biobank, and the Icelandic birth register)."<br>Materials and Methods, subsection "Summary data for outcomes":<br>"All included GWAS are from European ancestry samples to ensure similar pattern of linkage disequilibrium."
		ii. Provide information on the number of individuals who overlap between the exposure and outcome studies	It is not possible to know the number of overlapping samples. But we took this into account:<br>Materials and Methods, subsection "Mendelian randomization analysis", par. 4:<br>"Putative bias due to sample overlap between the exposure and outcome GWAS was quantified using the method proposed by Burgess and Davies (49)."<br>Results, subsection "Main Mendelian randomization analysis", par. 2:<br>"Assuming that the 217,397 samples from UKBB at the Birth weight GWAS are also among the 361,315 samples from UKBB at the depression GWAS, and that the 11,526 samples from Iceland at the depression GWAS are also among the 125,541 samples from Iceland at the Birth Weight GWAS, the maximum sample overlap is around 45% of the depression samples. Taking into the lower limit of the F-statistic 95% CI, 71.15, the estimated bias associated with overlap was negligible."
11	**Main results**		
	a)	Report the associations between genetic variant and exposure, and between genetic variant and outcome, preferably on an interpretable scale	Table 1 (exposure), Fig. 5.
	b)	Report MR estimates of the relationship between exposure and outcome, and the measures of	Fig. 3A.<br>Results, subsection "Main Mendelian randomization analysis", par. 1:

		uncertainty from the MR analysis, on an interpretable scale, such as odds ratio or relative risk per SD difference	"IVW method based on 14 trophoblast IVs was significant for depression after correction for multiple tests (beta = -0.165, 95% CI = -0.282 to -0.047, P = 0.0059)."
	c)	If relevant, consider translating estimates of relative risk into absolute risk for a meaningful time period	Not applicable.
	d)	Consider plots to visualize results (e.g., forest plot, scatterplot of associations between genetic variants and outcome versus between genetic variants and exposure)	Fig. 3A, Fig. 5.
12	**Assessment of assumptions**		
	a)	Report the assessment of the validity of the assumptions	Results, Subsection "Selection of SNPs as instrumental variables based on trophoblast-specific gene expression", par. 2:<br>"Individual F-statistic ranged from 48.30 to 165.37, indicative of no problems with weak instrument bias. The total number of IVs common to each exposure-outcome analysis was 13-15. The overall F-statistic ranged from 78.21 (depression) to 84.18 (ADHD). The mean F-statistic ranged from 78.63 (depression) to 83.99 (ADHD). The rest of the outcomes employ the same 15 IVs whose overall F-statistic value is 81.78 and mean F-statistic is 81.56."<br>Results Subsection "Sensitivity analysis for the birth weight – depression association", par. 1:<br>"Egger intercept was not significant (intercept = -0.006, 95% CI = -0.004 to -0.016, P = 0.24), suggesting absence of directional (or unbalanced) pleiotropy."
	b)	Report any additional statistics (e.g., assessments of heterogeneity across genetic variants, such as I^2, Q statistic or E-value)	Results Subsection "Sensitivity analysis for the birth weight – depression association", par. 1:<br>"In agreement with the absence of heterogeneity (Q = 9.07, P = 0.77), the association remained significant in leave-one-out analysis (Figure 4). Furthermore, MR-PRESSO did not detect any outlier using the default outlier significance threshold of 0.05. […] However, there is absence of heterogeneity (Q' = 7.514, P =0.82),

			and the difference Q – Q’ was not significant (1.556, P = 0.21), indicating that MR-Egger does not fit substantially better to the data.”
13	**Sensitivity analyses and additional analyses**		
	a)	Report any sensitivity analyses to assess the robustness of the main results to violations of the assumptions	Results Subsection “Sensitivity analysis for the birth weight – depression association”,<br>“The MR-Egger estimate was not significant (beta = 0.084, 95% CI = -0.324 to 0.493, P = 0.69), and the causal estimate is in the opposite direction (Figure 5). However, [...], indicating that MR-Egger does not fit substantially better to the data. The weighted median estimate was similar to the IVW (beta = -0.152, 95% CI = -0.313 to 0.009) (Figure 5). The result is near significance (P = 0.0638), in agreement with the low power of the method. Finally, the weighted mode ...”
	b)	Report results from other sensitivity analyses or additional analyses	Fig. 4, Fig. 5, Table S2.<br>Results Subsection “Sensitivity analysis for the birth weight – depression association”
	c)	Report any assessment of direction of causal relationship (e.g., bidirectional MR)	Fig. 3B.<br>Results, subsection “Main Mendelian randomization analysis”, par. 1:<br>“When birth weight was considered the outcome and each psychiatric disorder the exposure, there were no association, although PTSD was not tested due to lack of IVs (Figure 3B).”
	d)	When relevant, report and compare with estimates from non-MR analyses	Not applicable.
	e)	Consider additional plots to visualize results (e.g., leave-one-out analyses)	Leave-one-out analyses: Fig. 4<br>Scatterplot of SNP effects: Fig. 5
**DISCUSSION**			
14	**Key results**	Summarize key results with reference to study objectives	Discussion, par. 1:<br>“Using a MR approach, we present evidence for the involvement of placenta in susceptibility to broadly defined depression. Specifically, we selected SNPs with fetal effect on birth weight...”
15	**Limitations**	Discuss limitations of the study,	Limitations, par. 1:

		taking into account the validity of the IV assumptions, other sources of potential bias, and imprecision. Discuss both direction and magnitude of any potential bias and any efforts to address them	"The findings in this work must be interpreted in the context of a MR approach. The reliability of MR results is based on several IV assumptions. While the strength of the association between IVs and exposure was confirmed by the F-statistics…"<br>Limitations, par. 2:<br>"Another limitation was the low sample size of some of the GWAS of psychiatric disorders reducing the power of the analysis. ..."
16	**Interpretation**		
	a)	Meaning: Give a cautious overall interpretation of results in the context of their limitations and in comparison, with other studies	Discussion, par. 2:<br>"A previous MR study analyzed effects of birth weight on several psychiatric disorders, using a different approach, as their SNP effects were direct fetal effects adjusted for maternal genotype (12) …"<br>Discussion, par. 3:<br>"The outcome GWAS used for depression was based on a broad, highly heterogeneous, phenotype, which includes from people with self-reported depressive symptoms to patients ascertained with a diagnostic interview (40). [...]Thus, it may be possible that the causal association we detected is not specific to depression, but it was the only significant result in MR due to higher power. This agrees with the epidemiological study of Pettersson et al. (8), who found an association of birth weight with a general psychopathology factor."<br>Limitations, par. 1:<br>"The findings in this work must be interpreted in the context of a MR approach..."
	b)	Mechanism: Discuss underlying biological mechanisms that could drive a potential causal relationship between the investigated exposure and the outcome, and whether the gene-environment equivalence assumption is reasonable. Use causal language carefully, clarifying that IV	Discussion, par. 4:<br>"The causal effect of trophoblast functioning on susceptibility to psychopathology may be related to the process of invasion of the uterine walls that takes place in the early stages of placentation. The aim of this process …"<br>Limitations, par. 1:<br>"The findings in this work must be interpreted in the context of a MR approach. The reliability of MR results is based on several IV

		estimates may provide causal effects only under certain assumptions	assumptions. […] However, we cannot discard an effect of the selected IVs by another mechanism not related with fetal growth restriction.”
	c)	Clinical relevance: Discuss whether the results have clinical or public policy relevance, and to what extent they inform effect sizes of possible interventions	Conclusion:<br>“Although no treatments for fetal growth restriction are currently available, there are several promising agents on clinical trials or preclinical research, acting on processes such as oxygen supply by angiogenesis or vasodilatation, or mitigation of oxidative stress (69,70). Therefore, further research is needed to confirm this causal effect, as it could have clear implications in prevention of mental disorders in near future.”
17	**Generalizability**	Discuss the generalizability of the study results (a) to other populations, (b) across other exposure periods/timings, and (c) across other levels of exposure	Limitations, par. 2:<br>“Finally, the studies included in the PlacentalCellEnrich database (30) use expression data for placentas in the first three months of pregnancy. Although this period is key for establishment of the maternal-fetal interface, it would be necessary to include results from placentas in the second and third trimester to obtain a more complete view of placenta-associated variants.”
**OTHER INFORMATION**			
18	**Funding**	Describe sources of funding and the role of funders in the present study and, if applicable, sources of funding for the databases and original study or studies on which the present study is based	Section “Funding”.
19	**Data and data sharing**	Provide the data used to perform all analyses or report where and how the data can be accessed, and reference these sources in the article. Provide the statistical code needed to reproduce the results in the article, or report whether the code is publicly accessible and if so, where	Supplementary Table 2.
20	**Conflicts of interests**	All authors should declare all	Section “Conflict of Interest”.

		potential conflicts of interest	

Supplementary Table 2. Descriptive information on the GWAS used in the analysis.

Trait	N	N cases	N controls	Effective N[a]	Mean (s.d.)	Diagnostic criteria[b]	Reference
**Birthweight**	423,683				3,715 g (492)	Exclusion of multiple births, infant deaths and out-of-term births (gestational age <258 d and gestational age ≥294 d). Any extreme outliers in the birth weight. Babies born with congenital anomalies. UKBB only included individuals between 2.5 and 4.5 kg.	Juliusdottir et al. (5)
**Alcohol dependence**	38,686	10,206	28,480	30,054		Diagnose by DSM-IV (or DSM-IIIR) criteria derived either from clinician ratings or semistructured interviews.	Walters et al. (6)
**Attention-deficit/hyperactivity disorder**	225,534	38,691	186,843	128,213.8		Singletons diagnosed by psychiatrists according to the ICD10 criteria (F90.0, F90.1, F98.8 diagnosis codes) or individuals who had been prescribed medication specific for ADHD symptoms (ATC-NA06BA, mostly methylphenidate).	Demontis et al. (7)
**Autism spectrum disorder**	46,350	18,381	27,969	44,366.6		Singletons diagnosed with ASD by a psychiatrist according to ICD10, including diagnoses of childhood autism (ICD10 code F84.0), atypical autism (F84.1), Asperger's syndrome (F84.5), other pervasive developmental disorders (F84.8), and pervasive developmental disorder unspecified (F84.9).	Grove et al. (8)
**Bipolar disorder**	413,466	41,917	371,549	150,669.9		Required to meet international consensus criteria (DSM-IV, ICD-9 or ICD-10) for a lifetime diagnosis of BD, established using structured diagnostic instruments	Mullins et al. (9)

					from assessments by trained interviewers, clinician-administered checklists or medical record review.	
**Cannabis use disorder**	357,806	14,080	343,726	54,103.8	PGC cases met criteria for a lifetime diagnosis of DSM-IV (or DSM-III-R) cannabis abuse or dependence derived from clinician ratings or semi-structured interviews. iPSYCH sample had ICD-10 codes of F12.1 (cannabis abuse) or F12.2 (cannabis dependence), or both. deCODE sample met criteria for lifetime DSM-III-R or DSM-IV cannabis abuse or dependence or DSM-5 cannabis use disorder.	Johnson et al. (10)
**Major depressive disorder**	500,199	170,756	329,443	449,855.9	Three depression phenotypes were assessed ranged self-reported help-seeking for problems with nerves, anxiety, tension or depression (termed 'broad depression'), probable MDD based on self-reported depressive symptoms with associated impairment, and MDD identified from hospital admission records. Exclusions were applied to participants who were identified with bipolar disorder, schizophrenia, or personality disorder using self-declared data as well as prescriptions for antipsychotic medications.	Howard et al. (11)
**Post-traumatic stress disorder**	174,659	23,212	151,447	80,508.6	Diagnose was based either on lifetime (where possible) or current PTSD (i.e., including participants with a potential lifetime PTSD diagnosis as controls), and PTSD diagnosis was established using various	Nievergelt et al. (12)

						instruments and different versions of the DSM (DSM-III-R, DSM-IV, DSM-5).	
**Schizophrenia**	130,644	53,386	77,258	126,282		Consensus diagnosis (DSM-IV, ICD-10), research diagnostic interview, review of medical records, mixed strategy.	Trubetskoy et al. (13)

[a]Estimated as twice the harmonic mean.
[b]For further details see the original paper (Reference column).

Supplementary Table 3. Instrumental variables used in the MR analysis associated to GO term "embryonic placenta development".

SNP ID	Position hg38	Alleles[a]	EAF[b]	Beta (s.e.) birth weight	P birth weight	Gene in GO term	R^2	F-statistic
rs1374204	Chr2:46257066	C/T	0.2813	-0.0472(0.0028)	4.4E-64	*EPAS1*	9.24E-04	391.97
rs4953353	Chr2:46340137	T/G	0.3698	-0.0192(0.0026)	9.7E-14	*EPAS1*	1.71E-04	72.49
rs577204588[c]	Chr6:53156939	C/T	0.0010	-0.1535(0.0234)	5.8E-11	*GCM1*	2.48E-04	105.09
rs11042596	Chr11:2097630	T/G	0.3807	0.026(0.0027)	1.3E-22	*IGF2*	2.96E-04	125.56
rs1011476[c]	Chr11:2277805	T/G	0.2465	0.0179(0.0027)	4E-11	*ASCL2, IGF2*	1.30E-04	55.15
rs234864[c]	Chr11:2836067	G/A	0.4523	-0.0169(0.0025)	1.2E-11	*CDKN1C*	1.42E-04	60.22
rs4444073[c]	Chr11:10310117	C/A	0.4732	-0.0219(0.0024)	2.8E-19	*ADM*	2.39E-04	101.39
rs55836809	Chr13:27928737	G/A	0.2316	-0.0204(0.003)	1.9E-11	*CDX2*	1.41E-04	59.87
rs55958435	Chr15:96309409	G/A	0.2127	-0.0247(0.0028)	4.3E-18	*NR2F2*	2.32E-04	98.31
rs41355649[c]	Chr19:33299650	A/G	0.0646	-0.0366(0.0051)	5E-13	*CEBPA*	1.63E-04	69.09
rs1135856	Chr22:46044725	C/T	0.2684	-0.0192(0.0028)	5.6E-12	*WNT7B*	1.51E-04	63.81

[a]First allele is the effect allele
[b]Effect allele frequency in the 1000Genomes reference panel (European population)
[c]Common to main analysis.

2 References

1. Jain A, Tuteja G. PlacentaCellEnrich: A tool to characterize gene sets using placenta cell-specific gene enrichment analysis. Placenta (2021) 103:164–171. doi: 10.1016/J.PLACENTA.2020.10.029

2. Vento-Tormo R, Efremova M, Botting RA, Turco MY, Vento-Tormo M, Meyer KB, Park JE, Stephenson E, Polański K, Goncalves A, et al. Single-cell reconstruction of the early maternal–fetal interface in humans. *Nature 2018 563:7731* (2018) 563:347–353. doi: 10.1038/s41586-018-0698-6

3. Suryawanshi H, Morozov P, Straus A, Sahasrabudhe N, Max KEA, Garzia A, Kustagi M, Tuschl T, Williams Z. A single-cell survey of the human first-trimester placenta and decidua. *Sci Adv* (2018) 4: doi: 10.1126/SCIADV.AAU4788

4. Skrivankova VW, Richmond RC, Woolf BAR, Yarmolinsky J, Davies NM, Swanson SA, Vanderweele TJ, Higgins JPT, Timpson NJ, Dimou N, et al. Strengthening the Reporting of Observational Studies in Epidemiology Using Mendelian Randomization: The STROBE-MR Statement. *JAMA* (2021) 326:1614–1621. doi: 10.1001/JAMA.2021.18236

5. Juliusdottir T, Steinthorsdottir V, Stefansdottir L, Sveinbjornsson G, Ivarsdottir E V., Thorolfsdottir RB, Sigurdsson JK, Tragante V, Hjorleifsson KE, Helgadottir A, et al. Distinction between the effects of parental and fetal genomes on fetal growth. *Nature Genetics 2021 53:8* (2021) 53:1135–1142. doi: 10.1038/s41588-021-00896-x

6. Walters RK, Polimanti R, Johnson EC, McClintick JN, Adams MJ, Adkins AE, Aliev F, Bacanu SA, Batzler A, Bertelsen S, et al. Trans-ancestral GWAS of alcohol dependence reveals common genetic underpinnings with psychiatric disorders. *Nat Neurosci* (2018) 21:1656. doi: 10.1038/S41593-018-0275-1

7. Demontis D, Walters GB, Athanasiadis G, Walters R, Therrien K, Nielsen TT, Farajzadeh L, Voloudakis G, Bendl J, Zeng B, et al. Genome-wide analyses of ADHD identify 27 risk loci, refine the genetic architecture and implicate several cognitive domains. *Nature Genetics 2023 55:2* (2023) 55:198–208. doi: 10.1038/s41588-022-01285-8

8. Grove J, Ripke S, Als TD, Mattheisen M, Walters RK, Won H, Pallesen J, Agerbo E, Andreassen OA, Anney R, et al. Identification of common genetic risk variants for autism spectrum disorder. *Nat Genet* (2019) 51:431–444. doi: 10.1038/S41588-019-0344-8

9. Mullins N, Forstner AJ, O'Connell KS, Coombes B, Coleman JRI, Qiao Z, Als TD, Bigdeli TB, Børte S, Bryois J, et al. Genome-wide association study of more than 40,000 bipolar disorder cases provides new insights into the underlying biology. *Nat Genet* (2021) 53:817–829. doi: 10.1038/S41588-021-00857-4

10. Johnson EC, Demontis D, Thorgeirsson TE, Walters RK, Polimanti R, Hatoum AS, Sanchez-Roige S, Paul SE, Wendt FR, Clarke TK, et al. A large-scale genome-wide association study meta-analysis of cannabis use disorder. *Lancet Psychiatry* (2020) 7:1032–1045. doi: 10.1016/S2215-0366(20)30339-4

11. Howard DM, Adams MJ, Clarke TK, Hafferty JD, Gibson J, Shirali M, Coleman JRI, Hagenaars SP, Ward J, Wigmore EM, et al. Genome-wide meta-analysis of depression identifies 102 independent variants and highlights the importance of the prefrontal brain regions. *Nature Neuroscience 2019 22:3* (2019) 22:343–352. doi: 10.1038/s41593-018-0326-7

12. Nievergelt CM, Maihofer AX, Klengel T, Atkinson EG, Chen CY, Choi KW, Coleman JRI, Dalvie S, Duncan LE, Gelernter J, et al. International meta-analysis of PTSD genome-wide association studies identifies sex- and ancestry-specific genetic risk loci. *Nature Communications 2019 10:1* (2019) 10:1–16. doi: 10.1038/s41467-019-12576-w

13. Trubetskoy V, Pardiñas AF, Qi T, Panagiotaropoulou G, Awasthi S, Bigdeli TB, Bryois J, Chen CY, Dennison CA, Hall LS, et al. Mapping genomic loci implicates genes and synaptic biology in schizophrenia. *Nature* (2022) 604:502–508. doi: 10.1038/S41586-022-04434-5
